# Colonic Mucosal Epigenome and Microbiome Development in Children and Adolescents

**DOI:** 10.1155/2016/9170162

**Published:** 2016-02-23

**Authors:** R. Alan Harris, Rajesh Shah, Emily B. Hollister, Rune Rose Tronstad, Nils Hovdenak, Reka Szigeti, James Versalovic, Richard Kellermayer

**Affiliations:** ^1^Department of Molecular and Human Genetics, Baylor College of Medicine, Houston, TX 77030, USA; ^2^Section of Gastroenterology and Hepatology, Baylor College of Medicine, Houston, TX 77030, USA; ^3^Department of Pathology & Immunology, Baylor College of Medicine, Houston, TX 77030, USA; ^4^Texas Children's Hospital, Houston, TX 77030, USA; ^5^Department of Clinical Science, University of Bergen, 5020 Bergen, Norway; ^6^Department of Pediatrics, Haukeland University Hospital, 5021 Bergen, Norway; ^7^Department of Clinical Medicine, University of Bergen, 5020 Bergen, Norway; ^8^Section of Pediatric Gastroenterology, Department of Pediatrics, Baylor College of Medicine, Houston, TX 77030, USA; ^9^USDA/ARS Children's Nutrition Research Center, Houston, TX 77030, USA

## Abstract

Epigenetic and microbiome changes during pediatric development have been implicated as important elements in the developmental origins of inflammatory bowel diseases (IBDs) including Crohn's disease (CD) and ulcerative colitis (UC), which are linked to early onset colorectal cancer (CRC). Colonic mucosal samples from 22 control children between 3.5 and 17.5 years of age were studied by Infinium HumanMethylation450 BeadChips and, in 10 cases, by 454 pyrosequencing of the bacterial* 16S rRNA* gene. Intercalating age-specific DNA methylation and microbiome changes were identified, which may have significant translational relevance in the developmental origins of IBD and CRC.

## 1. Introduction

The epidemiology of inflammatory bowel diseases (IBDs) strongly implicates their environmental origin [1]. IBDs (Crohn's disease (CD) and ulcerative colitis (UC)) affect close to 1 in every 200 people just in the United States [2]. There has been a 5–10-fold increase in the prevalence of CD and a 2–10-fold increase in the prevalence of UC over the past 5-6 decades in developed countries [3, 4]. IBD incidence peaks in young adulthood, and its prevalence appears to be continuously rising in children [5, 6]. This epidemiology underscores the potential importance of the pediatric developmental period in the pathogenesis of IBD. While IBD is thought to be influenced by genetics, host immune dysfunction, mucosal barrier defects, and the gut microbiome [7], the exact etiology remains unknown [1].

High monozygotic twin discordance rates and other epidemiologic observations highlight the importance of nongenetic (such as epigenetic) processes, which are vulnerable to environmental (including nutritional) influences, in the etiology of IBD [1]. The increased incidence of IBD in populations migrating from low incidence to high incidence areas of the world [8] also supports this contention. These migration studies indicate that the prenatal and pediatric developmental periods are the most important in regard to the environmental developmental origins of IBD [1].

The potential importance for epigenetics in IBD is only recently receiving more attention [9]. There are many indications that epigenetic mechanisms other than DNA methylation (the methylation of cytosine at CpG dinucleotides) may contribute to the development, progression, and/or maintenance of IBD, such as histone modifications [10], and microRNAs [11]. However, only DNA methylation has been described to directly communicate environmental exposures to phenotypic outcome in mammals (reviewed in [1]).

The first example for nutritional imprinting via environmental exposure was described in the viable yellow Agouti (A^vy^) mouse [12]. A handful of similarly behaving mammalian genomic loci have been identified [13] and observations from us and others indicate that nutritionally sensitive, early-developmental epigenetic modifications are present in humans as well [14, 15]. We have recently expanded the compendium of such loci by utilizing colonic mucosal DNA from children with histologically normal colons (controls) [16]. We have also found that epigenetic shifts in mammalian colonic mucosa continue during the pediatric period, which may be relevant for age-dependent colitis susceptibility [17]. However, human colonic mucosal epigenetic plasticity during childhood and adolescence has not been assessed, especially in respect to potential age related microbiome-epigenome interactions. Such epigenetic plasticity in the large intestinal mucosa may have relevance to not only IBD but also early onset colorectal cancer (CRC), since IBD is a precancerous condition [18].

Here, we analyzed our recently published cohort of 22 control children between 3.5 and 17.5 years of age [19] in respect to age-dependent colonic mucosal DNA methylation and microbiome changes.

## 2. Materials and Methods

DNA isolated from left sided colonic mucosal samples was interrogated by Infinium HumanMethylation450 BeadChip Kits (Illumina San Diego, CA, USA). We determined beta values at each CpG site and selected those with significant age-dependent correlation (Spearman rank test, *p* < 0.05) and at least 10% methylation difference between the oldest and youngest proband. More specifically, transverse colonic mucosal biopsies were used for DNA extraction. Genomic DNA was isolated by bead beating and Qiagen TissueLyser [19] or by standard proteinase-k digestion and phenol-chloroform extraction as described previously [20]. DNA sample quality was examined with PicoGreen (https://www.promega.com/~/media/files/products%20and%20services/instruments/detection/tbs%20technical%20support%20docs/s-0041.pdf)before processing towards the microarrays. The samples that passed quality control were processed by Infinium HumanMethylation450 BeadChip Kits (Illumina San Diego, CA, USA; http://www.illumina.com/products/methylation_450_beadchip_kits.ilmn) according to the manufacturer's recommendations through automated processes in the Core Laboratory for Translational Genomics of the Baylor College of Medicine. Arrays were imaged with BeadArray Reader using standard Illumina scanner settings. The R Bioconductor minfi package [21] was used to generate beta values normalized to internal control probes. Internal controls determined the array processing to be of good quality. After removal of probes containing SNPs (http://www.rforge.net/IMA/snpsites.txt), 390,433 CpG probes on the array were used for subsequent analysis. Beta values at these CpG sites (according to GRCh37/hg19) were then correlated with the age of the 22 control proband. CpG sites with significant (Spearman rank test *p* < 0.05) correlation between DNA methylation and patient age (patients were between 3.5 and 17.5 years of age with grossly and histologically normal mucosa at colonoscopy; the indication for colonoscopy in these cases included abdominal pain, diarrhea, and hematochezia) were selected. Thereafter, only those CpG sites were included into the analysis of this report where 10% or more methylation difference between the oldest (17.5 years old) and youngest (3.5 years old) patient was present. To further increase the biological relevance of our findings we focused on genomic regions where at least 2 CpG sites met selection criteria within the same genomic region (an arbitrary cutoff of 15 kb was used as the “same genomic region”). DNA methylation at the CpG sites within these regions was examined in the transverse colonic mucosa of pediatric UC and CD patients. IBD dependent DNA methylation difference was examined by Student's *t*-test at these CpG sites. Those CpG sites were highlighted where *t*-test *p* value was less than 0.05.

In addition to the direct IBD associations, we examined links between the developmentally dynamic DNA methylation changes (described in Results) and the mucosal microbiome. This comparison was performed in a subset of 10 children and adolescents from the 22 controls. The DNA isolated from these patients' biopsy samples was analyzed not only by the bead-chip kits but by 454 pyrosequencing of the bacterial* 16S rRNA* gene as well. The analytical methods and microbiome results from these 10 controls were included in our prior publication examining the colonic mucosal microbiome of untreated pediatric CD patients [22]. However, the microbiome changes were not correlated with age in that work. Therefore, we performed correlation analysis (Pearson's correlation) between bacterial taxonomic abundance and patient age in transverse colonic mucosa and arbitrarily determined significance of *p* < 0.1. Therefore, this selection represents only trends and not false discovery rate corrected statistical correlations.

## 3. Results 

There were 621 CpG sites in somatic chromosomes where methylation decreased in an age-dependent (correlation with age *p* < 0.05) manner during childhood and adolescence according to our criteria (Supplementary Table 1 in Supplementary Material available online at http://dx.doi.org/10.1155/2016/9170162). DNA methylation increased at 852 sites with the same measures (Supplementary Table 2). Thereafter, we further delineated those genomic loci where at least 2 CpG sites met selection criteria within the same genomic region (an arbitrary cutoff of 15 kb was used as the “same genomic region”). The chance for such association to occur between 2 CpG sites is <10^−6^ (these regions are highlighted in Supplementary Tables 1-2). These genomic loci were arbitrarily defined as pediatric age-dependent differentially methylated regions (DMRs [23]). Twenty-three (23) such DMRs were detected with decreasing methylation during childhood and adolescence. Nineteen (19 = 82.6%) of these DMRs had direct gene associations according to the Illumina manifest. Eleven (11 = 57.9%) of these 19 genes have already been implicated in either colitis (*SHANK2, SLC9A3, TAGAP,* and* PON1*) or CRC (*KCNN3, SULT2B1, SP5, TRIM15, MLL3, CREB5,* and* SULF1*). Conversely, DNA methylation at 63 DMRs increased significantly during this pediatric developmental period. Thirty-seven (37 = 58.7%) of these DMRs associated with genes, but only 6 (16.2%) of those have been implicated in either colitis (*PTPRF, ELTD1,* and* GDNF*) or CRC (*PTPRF, ELTD1, PDX1, CCK,* and* AGPAT1*).

Importantly, we observed that average DNA methylation at 11 (47.8%) of the decreasing methylation DMRs was also significantly (*p* < 0.05) differentially methylated between UC and controls (Supplementary Table 3). In the meantime, only 3 (13%, 2 of which associated with genes) of these DMRs were significantly differentially methylated in the colonic mucosa of CD patients (Supplementary Table 4). Furthermore, 7 (70%) out of the 10 genes associated with the UC specific DMRs overlapping with age-dependent DNA methylation decline have already been observed to be differentially expressed in the colonic mucosa of adult UC patients [24], while none of the CD DMRs had such an association (Supplementary Table 3).

Among the 63 DMRs with increasing methylation during late pediatric development, 14 (22%; 8 of which associate with genes) were differentially methylated in pediatric UC patients (Supplementary Table 3), while only 4 (6.4%) were differentially methylated in CD patients (Supplementary Table 4). Three (37.5%) of the 8 genes associated with the UC DMRs in this group have been observed to be differentially expressed in the colonic mucosa of adult UC patients, while none of the CD DMRs had such an association.

To further highlight the biological relevance of the developmental DNA methylation changes identified herein, we compared our results to a recently published work on intestinal DNA methylation changes from fetal to pediatric development in humans [25]. This research group utilized the same conceptual approach as ours to define age-dependent intestinal epigenetic changes, which may be vulnerable to environmental influences thereby providing relevance to intestinal disease, such as IBD. Their age range of examination, however, was much broader than ours (fetal life 8–12 weeks of gestation to 12–14 years of age). Nevertheless, we found 5 shared CpG sites between our compendium (621 CpGs) and those CpG sites where DNA methylation highly significantly (corrected *p* for multiple testing <10^−7^) decreased between fetal and pediatric life (809 CpGs) (Supplementary Table 7). The chance for such overlap between the two compendiums to occur randomly is <10^−7775^. Four (80%) of the associated genes with these CpG sites have been linked to either ulcerative colitis (*IL4R* and* SAA1*) or CRC (*LAT2* and* SAA2*). In addition to shared CpG sites with decreasing methylation, we found 12 CpG sites where direct overlap between age-dependent increase in DNA methylation occurred between our compendium (852 CpGs) and that of Kraiczy et al. (1091 CpGs where DNA methylation between fetal and pediatric development significantly (corrected *p* < 10^−7^) increased) (Supplementary Table 7). The chance for such overlap between the two compendiums to occur randomly is <10^−2,000,000,000^. Five (42%) of the associated genes with these CpG sites have been linked to either ulcerative colitis (*FLT1* and* ELTD1*) or CRC (*PCDHG4A, GJD2,* and* TP53I11*). The shared CpG sites with age-dependent DNA methylation changes between our report (postnatal development) and the results of Kraiczy and colleagues (pre- and postnatal development) implicate that ~0.6–1% (5 out 809 and 12 out of 1091, resp.) of colonic mucosal epigenetic changes take place and/or proceed during childhood (beyond 3.5 years of age). Such developmentally plastic epigenetic processes are likely the most vulnerable to postnatal environmental (including nutritional) influences thereby providing a link between environment and colonic diseases affecting the mucosa [26]. Importantly, DNA methylation changes at* IL4R* [27] and* SAA1* [19] have been highlighted in the past to associate with UC, for example.

**Figure 1:**
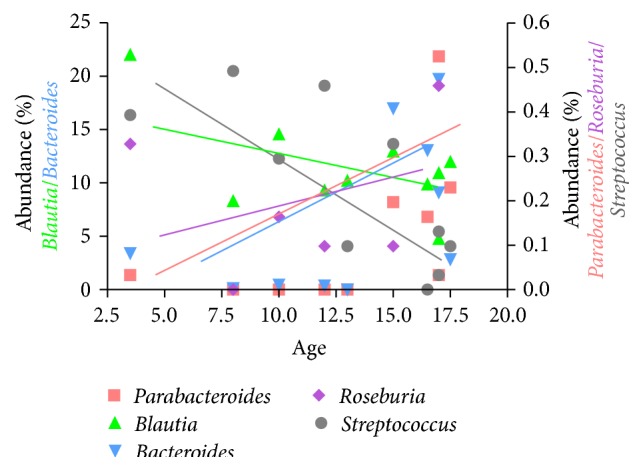
Significant (Pearson *p* < 0.1) correlations between colonic mucosal genera abundance and pediatric age. The abundance of* Streptococcus* (*r* = −0.75) and* Blautia* (*r* = −0.63) decreased, while that of* Parabacteroides* (*r* = 0.56),* Bacteroides* (*r* = 0.56), and* Roseburia* (*r* = 0.55) increased with age.

**Figure 2:**
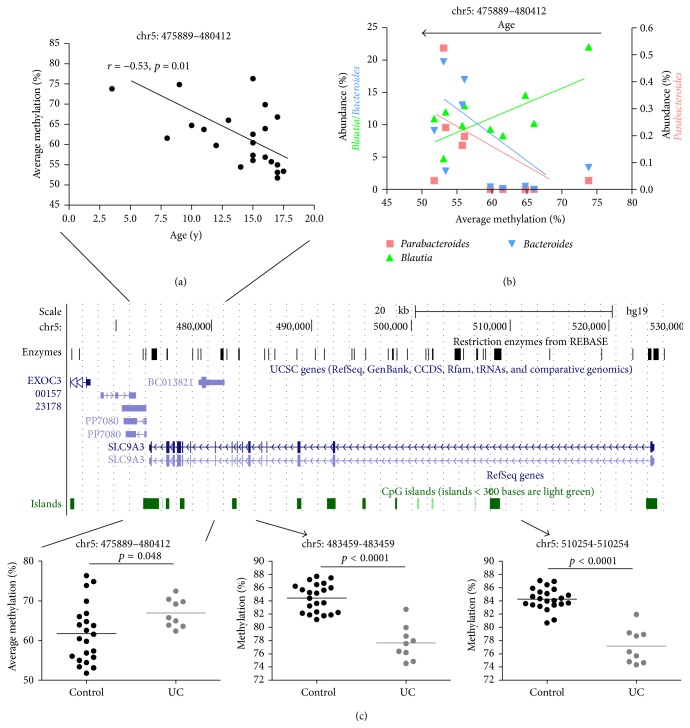
Epigenetic interactions with age, the microbiome, and ulcerative colitis (UC) at* SLC9A3*. Average methylation at 6 CpG sites within and surrounding the CpG island near the 3′ end of the gene significantly decreased with age (a). DNA methylation at this DMR significantly (Pearson *p* < 0.1) correlated with decreasing abundances of* Bacteroides* and* Parabacteroides* and increased abundance of* Blautia* (b). Methylation at the 3′ end DMR was significantly increased in UC patients, while it was decreased in 2 distinct upstream intragenic CpG sites (c), arguing for UC specific epigenetic rearrangement at* SLC9A3*, which is predicted to associate with decreased expression of the gene.

**Figure 3:**
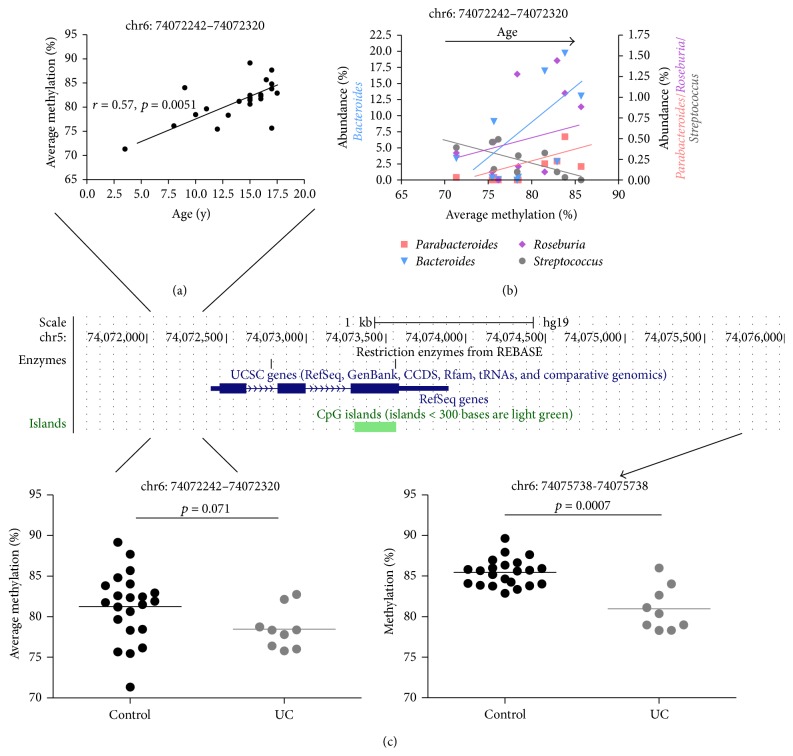
Epigenetic interactions with age, the microbiome, and ulcerative colitis (UC) at* KHDC3L* (*C6orf221*). Average methylation at 3 CpG sites in the promoter (lacking CpG island) region of the gene significantly decreased with age (a). DNA methylation at this DMR significantly (Pearson *p* < 0.1) correlated with decreasing abundance of* Streptococcus* and the increased abundance of* Bacteroides*,* Parabacteroides,* and* Roseburia* (b). Methylation of the promoter DMR tended to be decreased in UC patients (*p* = 0.071), along with significant decrease of methylation (*p* = 0.0007) at a distinct downstream CpG site in the 3′UTR (c), arguing for UC specific epigenetic rearrangement at this gene.

We then went on to examine age related pediatric microbiome variation in the large intestine and how that may correlate with epigenetic development in children. First, we determined if bacterial genera displayed age-specific variation with respect to abundance during childhood and adolescence. Here we relaxed the significance cutoff (Pearson correlation *p* < 0.1) secondary to the low number of samples available. The abundance of* Roseburia*,* Parabacteroides,* and* Bacteroides* increased with age (Figure 1). On the contrary, the abundance of* Streptococcus* and* Blautia* decreased during postinfantile pediatric development (Figure 1). Second, we determined age-dependent DMR correlation with the abundance of these genera. Only those DMRs were selected as having significant microbiome associations where DNA methylation in at least 2 CpG sites correlated significantly (*p* < 0.1) with the select genera abundance. Among the genes with decreasing methylation DMRs,* SLC9A3* and* PON1* had the highest number of genera (3/5 = 60%) correlations (Figure 2(b)). Among the genes with increasing methylation DMRs during pediatric development,* KHDC3L* (*C6orf221*) correlated with the greatest number of genera (4/5 = 80%) (Figure 3(b)). Conversely,* Roseburia* (Supplementary Table 5) and* Streptococcus* (Supplementary Table 6) were the genera with the highest number of correlations with epigenetically plastic DMRs during childhood and adolescence.

## 4. Discussion

We detected colonic mucosal epigenetic and metagenomic plasticity during late pediatric development, which demonstrated numerous significant interactions, highlighting the importance of both epidemiological and systems biology considerations when approaching the developmental origins of gastrointestinal diseases. We observed a remarkable link between age-dependent and IBD-specific DNA methylation variation, especially in respect to UC (significantly more developmentally shifting DMRs associated with UC than CD (two-tailed Fischer's exact *p* = 0.0007)) and DMRs with decreasing methylation (significantly more decreasing methylation DMRs correlated with UC than those with increasing methylation, *p* = 0.031) during late pediatric development. The compendium of DMRs outlined herein has functional relevance in respect to mammalian (including human) colitis and CRC based upon published mucosal gene expression and functional genomic data.

An outstanding example for the interactive epigenetic-metagenomic developmental plasticity is* SLC9A3* (encoding NHE3: sodium-hydrogen antiporter/exchanger 3, Figure 2). This gene has altered expression in the colonic mucosa of adult IBD patients [24, 28], and* SLC9A3* knockout mice develop colitis [29]. Importantly, these mice were observed to harbor a reduced-diversity microbiome, where* Bacteroides* and* Parabacteroides* abundances were significantly increased compared to wild type animals. Our results in humans would oppose these differences and indicate reduced* Bacteroides* in association with UC, based on the methylation pattern of* SLC9A3* (Figures 2(a)–2(c)). In fact Bacteroidales (the order into which both* Bacteroides* and* Parabacteroides* belong) was detected with decreased abundance in UC patients [30], consistent with our findings. In spite of these inconsistencies between humans and mice, an important interaction in mammals between mucosal* SLC9A3* and commensal bacteria appears to exist, which may be relevant for modulating colitis susceptibility.

Among the genes with increasingly methylated DMRs during pediatric development* KHDC3L* had the highest number of microbiome associations (Figures 3(a)–3(c)). This gene has been linked to hydatidiform mole formation [31]. Interestingly, we found a reported case of UC where the patient also developed vitiligo and hydatidiform mole [32]. We speculate that this case may highlight the potential role of* KHDC3L* in select UC patients, where genetic/epigenetic dysregulation at this gene could influence colonic mucosal pathology.

In respect to the further possible biologic relevance of our age-dependent metagenomic and epigenetic findings, we would like to highlight the numerous correlations between* Roseburia* abundance and epigenetically plastic DMR methylation changes during pediatric development.* Roseburia* are butyrate producing bacteria. Therefore, those can potentially modulate epigenetic changes in epithelial stem cells, since butyrate is a histone-deacetylase inhibitor (reviewed in [26]). In fact,* Roseburia* have been implicated to play a role in UC [33], for example.

The limitations of our study linking to small sample sizes and uncorrected *p* values in regard to significance are acknowledged. In the meantime, our work is the first to delineate age-dependent epigenetic and metagenomic changes in the large intestinal mucosa during human postnatal pediatric development. The selection and focus on genomic regions with at least 2 CpG sites meeting selection criteria (“DMRs”) increased the significance of our findings. Our previous work utilizing similar DMR criteria [16] has been deemed as overly conservative by a corresponding publication [34] in the field. The high degree of overlap between the selected genes and the already published literature on colitis and CRC support the biologic significance of the compendium in this work. This conclusion is further reinforced by several direct overlaps between the CpG sites with significant (although uncorrected *p* < 0.05) postnatal methylation changes in this report and the CpG sites from Kraiczy et al. [25] where highly significant (corrected *p* < 10^−7^) DNA methylation changes occurred from human fetal to pediatric development. This latter result validates the findings of both independent studies and indicates that epigenetic changes in human colonic mucosa take place and/or proceed during postnatal development, similarly as in mice [17].

We trust that the compendium of the developmentally plastic colonic mucosal DMRs identified herein will provide high impact targets for the biomedical field in respect to the generation of novel epigenetically and metagenomically focused preventative and therapeutic measures for both IBD and CRC.

## Availability of Supporting Information

Infinium HumanMethylation450 BeadChip data for colon mucosa samples are available through the NCBI Gene Expression Omnibus under accessions GSE32146 (https://www.ncbi.nlm.nih.gov/geo/query/acc.cgi?acc=GSE32146) and GSE42921 (https://www.ncbi.nlm.nih.gov/geo/query/acc.cgi?acc=GSE42921). The bacterial* 16S rRNA* sequences are available at NCBI SRA (BioProject: PRJNA284397).

## Supplementary Material

These tables (1–7) contain the developmentally shifting, and microbiome abundance linked CpG sites, which are highlighted within this publication.

## Abbreviations

CD: Crohn's disease
DMR: Differentially methylated region
IBD: Inflammatory bowel disease
UC: Ulcerative colitis.
